# A Large Pleural Effusion following Abdominal Aortic Surgery

**DOI:** 10.1155/2015/254010

**Published:** 2015-11-09

**Authors:** Vinoo K. Ramsaran, Vandana K. Seeram, James Cury, Adil Shujaat

**Affiliations:** Division of Pulmonary and Critical Care Medicine, University of Florida College of Medicine, Jacksonville, FL 32209, USA

## Abstract

Chylous ascites and coexistent chylothorax is a rare but important complication following retroperitoneal abdominal surgery. We report a 70-year-old male who developed gradual abdominal distension, chest tightness, and dyspnea five months after having an uncomplicated aortobifemoral bypass performed. Physical examination was consistent with a large right sided effusion and ascites which were confirmed by computed tomography. Thoracentesis yielded an opaque milky fluid with analysis consistent with a chylothorax with a paracentesis revealing fluid that was similar in both appearance and biochemistry. The patient failed initial conservative management so a chest tube was placed followed by chemical pleurodesis. We review the literature of the pathophysiology and treatment approach to such a pleural effusion.

## 1. Case Presentation

A 70-year-old man with chronic obstructive pulmonary disease (COPD) was referred to the hospital by his primary care physician (PCP) after 2 weeks of worsening dyspnea on minimal exertion, associated with a feeling of “fullness” in his chest. He denied chest pain, wheezes, discolored phlegm, hemoptysis, orthopnea, and ankle edema but complained of fifteen-pound weight loss despite an unchanged appetite and an increase in the size of his belly. His PCP obtained a chest radiograph and referred him to the hospital for further evaluation and management.

He was a hypertensive, ex-smoker of 180 pack years and an avid beer drinker of twelve beers daily but stopped both five months ago when he underwent aortobifemoral bypass grafting for incapacitating claudication.

Physical examination revealed a thin gentleman with poor dentition and oxygen saturations of 96% on room air. Chest exam revealed dullness in the lower half of his right hemithorax with absent breath sounds and decreased vocal and tactile fremitus consistent with a large right sided pleural effusion. Abdominal examination revealed distension with fullness in his flanks and shifting dullness consistent with ascites. There were no stigmata of chronic liver disease, clubbing, or ankle edema.

**Figure 1:**
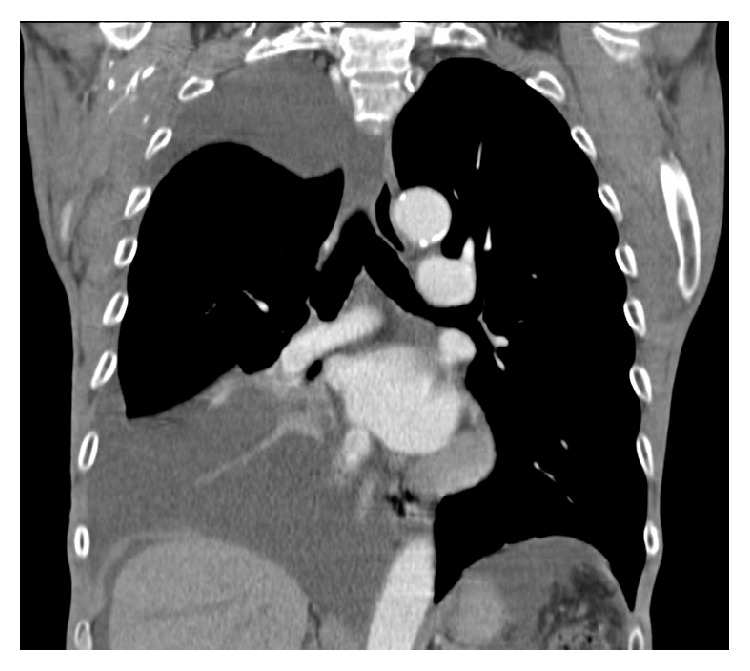
CT scan of the chest with intravenous contrast (coronal view) showing large right sided pleural effusion with lower lobe atelectasis and perihepatic ascites.

Complete blood count with differential, basic metabolic panel and hepatic panel was unremarkable except for a normocytic anemia (hemoglobin 9.2 g/dL) and hypoalbuminemia (serum albumin 3.2 g/dL). N-Terminal Pro-Brain Natriuretic Peptide was within normal limits. The chest radiograph done prior to this hospital visit reportedly showed a large right sided pleural effusion. A computed tomography (CT) scan of chest done on presentation to the hospital confirmed the large right sided pleural effusion (Figure 1) and also showed ascites.

**Figure 2:**
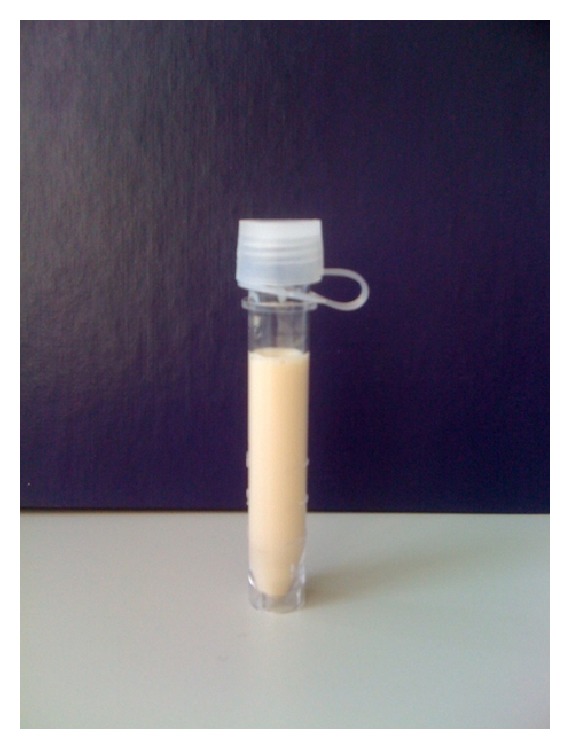
Pleural fluid obtained on thoracentesis.

A thoracentesis was performed and approximately 1500 mL of opaque milky fluid was obtained (Figure 2). Pleural fluid to serum LDH ratio was 1.6 consistent with the fluid being an exudate. The cell count and differential showed a white blood cell count (WBC) of 180/*μ*L with a lymphocytic predominance (79%). The pleural fluid cholesterol was 52 mg/dL with a triglyceride level of 1135 mg/dL. The fluid Gram stain was unremarkable and bacterial culture was sterile. A paracentesis was subsequently done and it also revealed a similar appearing fluid with a triglyceride level of 5010 mg/dL. This was consistent with chylous ascites with coexistent chylothorax.

## 2. Clinical Course

Vascular surgery was consulted for consideration of ligation of the cysterna chyli and its tributaries in the abdomen that were likely damaged at the time of the aortobifemoral bypass surgery and they recommended conservative management. The patient was placed on a medium chain triglyceride (MCT) diet which consisted of a low-fat diet supplemented with medium chain fatty acids in the form of caprylic and capric acids and subcutaneous octreotide 100 micrograms three times daily.

Despite conservative management, the pleural fluid reaccumulated and he developed dyspnea again. Although most cases of post-op chylous ascites respond to conservative measures [1], our patient also had a chylothorax and underlying COPD and remained too dyspneic to wait for the injury to heal, so a chest tube was placed and approximately 1250 cc of chylous fluid drained. In the interim, a peripherally inserted central venous catheter was inserted and TPN was initiated and his oral diet was stopped temporarily. Vascular surgery was reconsulted for surgical repair of the injury. However, they declined to operate on him.

Over the subsequent 4 days the output from his right sided chest tube became significantly less with only additional 550 cc drainage. On the fifth day after chest tube placement, chemical pleurodesis of his right hemithorax was performed using Bleomycin through the chest tube. His chest tube output decreased substantially over the next three days and his chest tube was removed.

**Figure 3:**
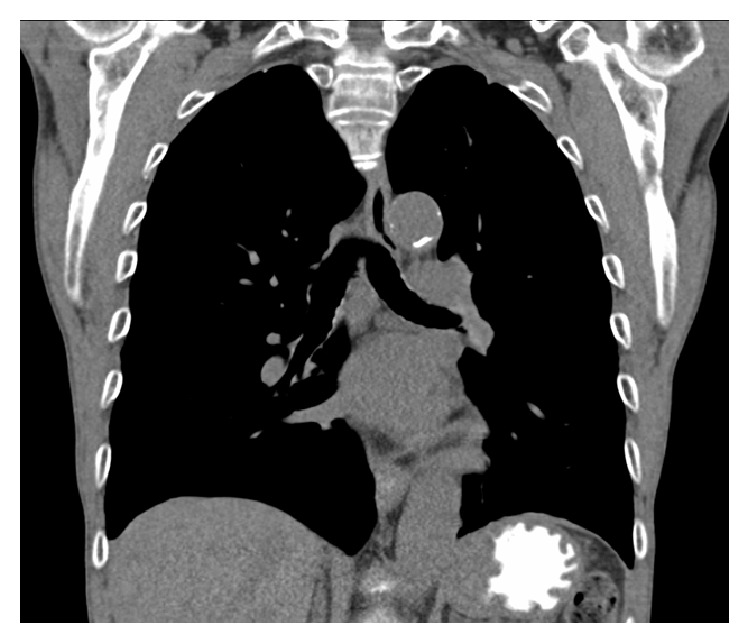
CT scan of the chest without intravenous contrast (coronal view) showing complete resolution of the right sided pleural effusion.

A low-fat diet with MCT supplementation was slowly reinitiated and he was discharged 1 month after his initial presentation. He was transferred to a skilled nursing facility where his MCT diet was slowly titrated up as his TPN was weaned off. He was seen for outpatient follow-up in the pulmonary clinic. At that time he had returned to his baseline and a CT scan of his chest performed by his PCP 2 weeks after discharge revealed complete resolution of the right pleural effusion and ascites (Figure 3).

## 3. Discussion

Chylous ascites and coexistent Chylothorax is a rare but important complication following retroperitoneal abdominal surgery [2–5], the incidence of which remains largely unknown. The largest series of 302 chylous effusions found that 33 (11%) were coexistent chylous ascites and chylothorax [5]. Patients with chylothorax have mortality rates approaching 50% depending on the underlying pathological condition in addition to an increased susceptibility to infection owing to a combination of lymphocyte loss in the chylous fluid and malnutrition as a result of protein, fat, and electrolyte depletion [6].

The diagnosis of chylothorax is usually made based on the clinical scenario, the gross appearance, and biochemical analysis of the pleural fluid. Chyle is a white, milky, and odorless fluid. There can be variations in the gross appearance of the fluid, however, ranging from bloody to turbid or even clear yellow in those with no oral intake which is sometimes the case after abdominal surgery. Pleural fluid analysis usually yields an exudate with a triglyceride level above 110 mg/dL (1.24 mmol/L), a ratio of the pleural fluid to the serum triglyceride of greater than 1.0, and a ratio of the pleural fluid to the serum cholesterol of less than 1.0 [7].

Chylous ascites can occur from operative trauma to the cisterna chyli or its tributaries or the thoracic duct. Chylothorax in the setting of chylous ascites is an example of a porous diaphragm syndrome with the movement of fluid through diaphragmatic pores or defects, similar to how a hepatic hydrothorax develops [8]. It was felt that this was the etiology of the chylothorax in our case because paracentesis also revealed chylous fluid. Consequently, the treatment approach to such a chylothorax consists of measures to reduce the formation of ascitic fluid, to prevent the movement of ascitic fluid across the diaphragm, and to drain or obliterate the pleural space. Also important is the replenishment of major nutritional losses.

The main goal of treatment is to decrease the production of chyle and its flow in the mesenteric lymphatics. The use of a low-fat diet with medium chain triglyceride (MCT) supplementation reduces the production and flow of chyle [9] and chylous effusions respond to an initial approach with a high-protein and low-fat diet with MCT in between 60% and 67% of cases [10]. MCT bypass the lymphatic glands and are absorbed into the portal venous system directly, as opposed to long chained triglycerides that pass through the mesenteric lymphatics and can increase lymph flow up to 200-fold [11]. Total parenteral nutrition (TPN) is used for patients who cannot tolerate oral nutrition or as a second line of treatment when ascites is refractory to dietary changes [11]. TPN also restores nutritional and metabolic deficits. The reported success rate varies, however, from 60% to 100% [1].

Continuous intravenous high dose somatostatin for postoperative lymphatic leaks was first reported as an effective way of reducing chyle formation in 1990 [12]. Since then there have been numerous reports confirming the efficacy of somatostatin or its subcutaneous analog octreotide [13–16]. The exact mechanism by which somatostatin works is unknown, but it has been shown to decrease the intestinal absorption of fats and also decrease triglyceride concentration in the thoracic duct chyle [17]. To date there have been no trials validating the use of octreotide for chylous ascites or chylothorax. Some authors suggest increasing the dose of octreotide after initiation but this was not done in our patient. Orlistat, which prevents lipase from digesting fat, has also been used [18]. Most cases of postoperative chylous ascites respond to conservative measures and surgical repair is usually only performed after a 4-week trial of such conservative management [1]. Peritoneovenous shunting is reserved as a secondary intervention for cases that do not respond to dietary intervention and surgical repair is not an option [11].

Thoracentesis and paracentesis may relieve symptoms of dyspnea and abdominal discomfort. However, chyle reaccumulates if measures are not taken to control its formation or in some cases despite such measures. Chest tube placement may provide constant relief from dyspnea but can lead to depletion of protein stores and also significantly impact the immune system by causing lymphopenia. Therefore, in cases where chyle reaccumulates in the pleural space too rapidly despite conservative measures and surgical repair of the injury in the abdomen is not an option, consideration should be given to obliterating the pleural space. Pleurodesis has been reported to be effective for recurrent chylothorax. One series of medical thoracoscopy and talc pleurodesis of 24 hemithoraces in 19 patients with chylothorax due to lymphoma reported a success rate of 100 percent at 30, 60, and 90 days [19]. Another retrospective study reported an 80% success rate using talc pleurodesis alone for patients with chylothorax resulting from various causes [20].

## 4. Conclusion

Chylous ascites with chylothorax is a rare complication of retroperitoneal surgery, particularly abdominal aortic surgery. Most cases respond to measures to reduce formation of chyle such as substitution of MCT for fat in the diet, somatostatin, orlistat, and TPN. Although thoracentesis provides rapid relief from dyspnea, the fluid reaccumulates. Chest tube drainage for relief of dyspnea is not recommended for prolonged period of time because it leads to malnutrition and immune deficiency. Most cases of postoperative chylous ascites respond to conservative measures and a trial of such measures is warranted. In cases where there is also a chylothorax and it reaccumulates too rapidly and causes significant dyspnea despite conservative measures, surgical repair or pleurodesis should be considered.
